# Maternal Anxiety Symptoms, Sensory Processing, and Play Performance in Children Aged 4–6 Years with Autism Spectrum Disorder: A Cross-Sectional Study

**DOI:** 10.3390/children13101401

**Published:** 2026-10-14

**Authors:** Sümeyye Akçay, Arzu Dinç Yavaş, Hatice Gamze Poyrazoğlu, Nil Su Cesar, İkra Nur Kaplan, Cevriye Ceyda Uğur

**Affiliations:** 1 Department of Occupational Therapy, Faculty of Health Sciences, Fenerbahçe University, Ataşehir, İstanbul 34758, Türkiyeikranurkaplan83@gmail.com (İ.N.K.);; 2 Department of Physical Medicine and Rehabilitation, Faculty of Medicine, İstanbul Aydın University, Küçükçekmece, İstanbul 34295, Türkiye; 3 Department of Pediatric Neurology, Faculty of Medicine, İstanbul Aydın University, Küçükçekmece, İstanbul 34295, Türkiye

**Keywords:** autism spectrum disorder, maternal anxiety symptoms, sensory processing, play, playfulness

## Abstract

**Highlights:**

**What are the main findings?**

• Maternal anxiety symptoms were linked to children’s play performance, playfulness, and specific sensory-processing patterns (low registration, sensation-seeking).
• Developmental play performance was independently associated with maternal anxiety symptoms, while sociodemographic factors were not; a preliminary association with comorbid ADHD (*n* = 10) requires confirmation.

**What are the implications of the main findings?**

• Family-centered occupational therapy assessment should jointly consider children’s play/sensory profile and maternal psychological wellbeing.
• Screening for maternal anxiety symptoms may be particularly warranted when a child has lower play performance.

**Abstract:**

**Background:** Children with autism spectrum disorder (ASD) often show atypical sensory processing and reduced play participation, and their mothers frequently report elevated anxiety. However, maternal anxiety symptoms, sensory processing, and children’s play performance and playfulness have rarely been examined together, and the factors independently associated with maternal anxiety symptoms have not yet been clarified. **Objective:** This study examined relationships between maternal anxiety symptoms and children’s sensory processing, play performance, and playfulness in children with ASD aged 4–6 years, and identified factors independently associated with maternal anxiety symptoms. **Method:** In this cross-sectional study, 58 mother–child dyads were recruited from a special education center in Istanbul, Türkiye. Maternal anxiety was assessed with the Beck Anxiety Inventory, sensory processing with the Dunn Sensory Profile, and play with the Revised Knox Preschool Play Scale and Test of Playfulness. **Results:** Maternal anxiety was negatively correlated with play performance (ρ = −0.564, *p* < 0.001) and playfulness (ρ = −0.492, *p* < 0.001), and positively correlated with low registration and sensation-seeking raw scores (ρ = 0.474, *p* < 0.001 and ρ = 0.343, *p* = 0.008, respectively; higher raw scores indicate more typical, less atypical processing). Children with comorbid ADHD had mothers with higher anxiety and lower play scores. In a multivariable model (R^2^ = 0.516), play performance (β = −0.411, *p* = 0.002) and, preliminarily, comorbid ADHD (β = 0.274, *p* = 0.027; only 10 children with ADHD) were independently associated with maternal anxiety; maternal education, employment, and school type were not. **Conclusions:** In young children with ASD, maternal anxiety symptoms were most strongly associated with the child’s play performance, while a preliminary association with co-occurring ADHD requires confirmation in larger samples; these findings support considering maternal wellbeing in family-centered occupational therapy assessment.

## 1. Introduction

Autism spectrum disorder (ASD) is a neurodevelopmental condition characterized by differences in social communication and by restricted, repetitive behaviors or interests [1]. Sensory-related features, including sensory hyperresponsiveness, commonly co-occur with these behaviors and with anxiety, and may make everyday environmental experiences particularly challenging [2]. In early childhood—a period consequential for later brain development, wellbeing, and functioning—these characteristics may shape children’s engagement in meaningful daily occupations [3]. Preschool-aged children with ASD have been observed to participate less diversely in play, physical recreation, and social activities than typically developing peers [4], with sensory-processing patterns separately linked to reduced participation in daily occupations [5]. From an occupational therapy perspective, however, participation is not determined by child characteristics alone: it emerges through interactions among the child’s abilities and sensory experiences, family support, activity demands, and the social and physical environment [6,7,8]. Since factors at both the child and family levels can jointly shape participation during this developmentally important period, the family context surrounding the participation of young children with ASD in play activities requires closer examination.

Sensory processing refers to how individuals detect, organize, modulate, and respond behaviourally to sensory information encountered in everyday life. Dunn’s framework conceptualizes this as four interacting patterns—low registration, sensation-seeking, sensory sensitivity, and sensation avoiding—arising from the relationship between neurological thresholds and self-regulation strategies, with individuals exhibiting varying degrees of each pattern rather than a single sensory type [9]. Sensory differences in ASD are frequently reported across auditory, visual, tactile, vestibular, proprioceptive, and oral domains, with one review reporting atypical sensory processing in 82–97% of autistic participants [10], and links to adaptive behaviour, attention, and participation in daily occupations reported across other studies using Dunn’s framework, though the direction of these relationships is often unclear [5,11,12]. Sensory reactivity is included among DSM-5 ASD diagnostic features, but an individual sensory profile is neither unique to autism nor inherently pathological, and its functional significance depends on the child, the activity, and the environment [11,12]. Sensory processing is therefore best considered a multidimensional, context-sensitive pattern rather than a single general deficit.

Play is a primary childhood occupation through which young children practice social-emotional, cognitive, language, self-regulatory, and executive-function skills in interactions with caregivers and peers [13]. In preschool children with ASD, play performance may differ from that of typically developing peers in the complexity of symbolic or pretend play and in spontaneous peer interaction [14,15]. From an occupational therapy perspective, however, play performance and playfulness are related but distinct constructs. Developmental play performance concerns what the child does during play—its observable organization, content, and social participation—and can be profiled using the Revised Knox Preschool Play Scale, which has demonstrated reliability and concurrent validity for preschoolers with ASD [16]. Playfulness concerns the manner or quality of engagement: Bundy’s framework emphasizes intrinsic motivation, perceived control, freedom from reality, and social cues, and the Test of Playfulness provides an observational measure of this construct [17]. Evidence indicates that the Test of Playfulness and Children’s Playfulness Scale converge in therapist ratings, while parent and therapist ratings may differ, underscoring that playfulness is context- and observer-sensitive rather than reducible to developmental skill level [18]. A child’s play performance and playfulness should therefore be assessed and interpreted separately.

The relationship between sensory processing and play in autistic children does not appear to operate through a single, uniform pathway, but depends on how sensory differences interact with a child’s praxis, attention, and activity demands. Autistic children have shown lower developmental play scores than peers, with associations between specific sensory patterns and play described as complex rather than uniform [19]; modelling suggested sensory-system function relates to play primarily through praxis—an indirect rather than a direct pathway [20]. A related indirect pathway has been reported outside the play literature specifically: emotion dysregulation has been shown to mediate the relationship between sensory processing and behavioral problems more broadly in young autistic children [21], raising the possibility that emotion regulation may represent a further mechanism worth examining for play outcomes specifically. Motor-planning difficulties have been linked to lower caregiver-rated play participation [22], and greater sensory differences to more atypical social attention during play-relevant tasks [23]. These associations are not deterministic, however: naturalistic observation has found no group differences in basic toy exploration under supportive, unstructured conditions [24], and qualitative accounts indicate that environmental features can both hinder and support participation depending on activity structure [25]. Therefore, sensory processing appears to shape not whether autistic children play, but rather how they engage in play.

Raising a child with ASD has consistently been associated with elevated maternal psychological burden, although the evidence is stronger for elevated distress in general than for any single causal pathway. A systematic review and meta-analysis of 31 studies estimated a median prevalence of clinically significant anxiety disorders of 33% among parents, with substantial heterogeneity across studies [26], and clinical samples have reported markedly higher rates of maternal anxiety symptoms, alongside associations with child social impairment [27]. Child-related factors are consistently linked to maternal distress: child behavior problems have been associated with maternal anxiety through parenting stress and lower parenting self-efficacy [28]. Longitudinal evidence, however, complicates a simple child-difficulties-cause-maternal-anxiety model: a population-defined cohort found that maternal distress and lower wellbeing predicted later child behavior problems, while early child behavior problems did not predict later maternal wellbeing [29], and other multi-wave studies have found reciprocal, or parent-driven rather than purely child-driven, associations that vary by domain [30]. Maternal wellbeing is also shaped by the broader caregiving context, with service continuity, professional involvement, and household income all implicated [31]. Maternal anxiety in this population is therefore best understood as embedded within a child–family–service-system transaction rather than as a simple, unidirectional consequence of the child’s difficulties.

While there are separate studies linking children’s sensory processing to their participation in daily occupations [5] and, in particular, their play performance [19,20]—as well as studies associating maternal anxiety (or parental distress more broadly) with children’s behavioural and functional difficulties [26,27,28]—research simultaneously addressing maternal anxiety, children’s sensory processing, and children’s play performance and playfulness within a single model remains limited. Loh et al. (2023), for example, examined sensory processing and participation across childhood occupations in autistic children but did not incorporate a caregiver-level psychological variable such as maternal anxiety in the same analysis [32]. Consequently, it remains unclear whether maternal anxiety relates independently to children’s play outcomes once child-level clinical and sociodemographic factors—comorbid ADHD, school type, and maternal education and employment status—are taken into account. Addressing this gap may help clarify which factors are most relevant to family-centered, occupation-based intervention planning in early childhood ASD.

The aim of this study was to examine the relationships between maternal anxiety symptoms and the sensory processing profile, developmental play performance, and playfulness of children with ASD aged 4–6 years, and to identify factors independently associated with maternal anxiety symptoms after accounting for relevant child and family characteristics. We further hypothesized that, among candidate child- and family-related variables—comorbid ADHD, school type, maternal education level, maternal employment status, and developmental play performance—at least one would be independently associated with maternal anxiety in a multivariable model.

## 2. Materials & Methods

### 2.1. Study Design

This study was designed as a cross-sectional observational study. The study protocol was approved by the Fenerbahçe University Non-Interventional Clinical Research Ethics Committee (protocol no: 37.2026fbu; date: 7 April 2026), and no participant screening, assessment, or data collection took place prior to this approval. The study was conducted in accordance with the principles outlined in the Declaration of Helsinki. Written informed consent was obtained from all participating mothers prior to data collection.

### 2.2. Sample Size

The sample size was determined using an a priori power analysis performed with G*Power version 3.1. Since the study was designed to examine the relationships among the main study variables, correlation analysis was selected, and a two-tailed test was used. The significance level was set at α = 0.05, and statistical power was set at 80%.

The expected effect size was based on the correlation coefficient reported by Loh et al. (2023), who examined the relationship between sensory processing and participation in children with autism spectrum disorder (r = 0.36) [32]. Based on these parameters, the minimum required sample size was calculated as 58 participants. Although the initial plan was to recruit 65–70 participants to account for possible missing data, the final analysis was completed with 58 participants, which met the minimum required sample size.

This power analysis was designed to ensure adequate power for the primary bivariate correlations between maternal anxiety and children’s sensory processing, play performance, and playfulness. Because a study-specific effect size estimate for the association between maternal anxiety and children’s sensory or play characteristics was not available in the literature, the anticipated correlation coefficient was drawn from Loh et al. (2023) [32], who examined sensory processing and participation rather than maternal anxiety specifically; this proxy estimate should therefore be regarded as an approximation rather than a direct empirical basis for the present associations. This calculation does not, by itself, establish adequate statistical power for the multivariable regression model, which included five predictors, or for the subgroup comparisons reported in the Section 3. The regression and subgroup analyses are accordingly interpreted as descriptive of this sample rather than as independently powered analyses, and the corresponding findings—particularly those involving the small ASD + ADHD subgroup (*n* = 10)—should be regarded as preliminary pending confirmation in independently powered, larger samples.

### 2.3. Participants

Children diagnosed with autism spectrum disorder who were receiving services at ATA Special Education and Rehabilitation Center in Istanbul, Türkiye, and their mothers were screened for eligibility between 13 April 2026 and 17 June 2026, using a convenience sampling approach in which children who were accessible at the center during this period and whose mothers were willing to participate were enrolled. The study sample consisted of mother–child dyads in which the child was aged between 4 and 6 years and had a diagnosis of autism spectrum disorder. ASD diagnoses, and comorbid ADHD diagnoses where present, had been established by a physician prior to the child’s referral to the rehabilitation center; children were enrolled in the study with their pre-existing clinical diagnoses, which were confirmed from the center’s clinical records rather than reassessed using a study-specific diagnostic instrument. Inclusion and exclusion criteria are presented in Table 1. ADHD is a common neurodevelopmental comorbidity of ASD and was not considered to fall within the “severe neurological, genetic, or psychiatric disorder” exclusion criterion, which was intended to exclude children with additional major diagnoses that could substantially confound the study’s focus on ASD-related sensory and play characteristics. ADHD was the only comorbid diagnosis present among the children included in the final sample. A formal log of families approached, screened, and declining participation was not maintained during the study period; consequently, the exact number of eligible families who did not participate, and the specific reasons for non-participation, cannot be reported. In the final analysis, 58 mother–child dyads who met the eligibility criteria were included.

### 2.4. Outcome Measurements

All outcome measurements were performed at a single time point after enrollment. Maternal anxiety was assessed using the Beck Anxiety Inventory. Children’s sensory processing profile was evaluated using the Dunn Sensory Profile, including low registration, sensation-seeking, sensory sensitivity, and sensation avoiding patterns. Play-related outcomes were assessed using the Revised Knox Preschool Play Scale and the Test of Playfulness. Demographic and clinical characteristics, including child age, sex, school type, comorbid diagnosis, previous therapy history, maternal age, maternal education level, maternal occupation, and maternal employment status, were also recorded.

The Dunn Sensory Profile and the play-related assessments were administered within the same overall assessment process, on the same day. The Beck Anxiety Inventory and the Dunn Sensory Profile, both completed by the mother, were collected by a different evaluator than the one who conducted the child’s play observation; all evaluators were occupational therapists. The play observation was conducted in a dedicated evaluation room within the clinic and lasted approximately 40 min. The observation followed a semi-structured format: rather than a fully unstructured free-play setting, a standard set of materials was presented to the child for the purpose of eliciting and observing play and interaction behaviors, including kinetic sand, Lego blocks, a foam ball, coloring materials, and matching cards, among others. The Revised Knox Preschool Play Scale and the Test of Playfulness were both scored from this single play observation. All 58 play observations were conducted and scored by the same evaluator, a recently graduated occupational therapist; sessions were not video- or audio-recorded, and each child was assessed in a single session. The evaluator’s administration and scoring of the Revised Knox Preschool Play Scale and the Test of Playfulness were based on standard occupational therapy training; no scale-specific certification, additional training, or calibration exercise was completed prior to data collection. No adults or children other than the evaluator, the child, and, for some participants, the mother were present during the observation. The evaluator who conducted the child’s play observation was blind to the mother’s Beck Anxiety Inventory score and to the child’s comorbid ADHD status. The mother’s presence in the room during the observation varied across participants; the exact number of assessments conducted with and without the mother present was not systematically recorded.

### 2.5. Demographic and Clinical Characteristics

Child-related demographic and clinical characteristics, including age (recorded in completed years rather than exact months), sex, school type, comorbid diagnosis, and previous therapy history, were recorded using a structured data form at enrollment. Maternal characteristics, including age, education level, occupation, and employment status, were also recorded. The comorbid diagnosis variable was coded according to the presence or absence of an additional diagnosis accompanying autism spectrum disorder.

### 2.6. Beck Anxiety Inventory

Maternal anxiety was assessed using the Beck Anxiety Inventory. The Beck Anxiety Inventory is a self-report measure developed to assess anxiety symptoms. The scale consists of 21 items, and each item is scored from 0 to 3, with higher total scores indicating greater anxiety symptom severity [33]. The Turkish version of the Beck Anxiety Inventory has been shown to have acceptable psychometric properties [34].

### 2.7. Dunn Sensory Profile

Children’s sensory processing characteristics were assessed using the Dunn Sensory Profile. The scale evaluates children’s responses to sensory stimuli and sensory processing patterns in daily life. It is based on Dunn’s sensory processing model and includes four main sensory processing patterns: low registration, sensation-seeking, sensory sensitivity, and sensation avoiding. The scale is evaluated using subscale scores rather than a total score. Each item is rated on a 5-point frequency scale (1 = always, 5 = never); because items describe potential sensory-processing difficulties, both the four main pattern scores and the 14 more specific domain scores are calculated as the sum of these item ratings, and lower scores indicate a higher frequency of the described difficulty—i.e., the corresponding pattern or domain is more prominent—whereas higher scores indicate less frequent occurrence, i.e., more typical processing [35]. The Turkish adaptation of the Sensory Profile, a 125-item caregiver questionnaire for children aged 3–10 years, was conducted by Kayıhan et al. [36]; its validation sample consisted of children with autism aged 5–10 years. The four patterns follow the terminology of Dunn’s model (low registration, sensation-seeking, sensory sensitivity, and sensation avoiding), and the 14 subsections (A–N) of the instrument are referred to as domains in this paper, using the original subsection names. Raw scores were analyzed; they were not converted to the instrument’s classification categories (typical performance, probable difference, and definite difference).

### 2.8. Revised Knox Preschool Play Scale

Children’s play skills were assessed using the Revised Knox Preschool Play Scale. This observational assessment tool is used to evaluate preschool children’s play skills. The scale includes four main domains: space management, material management, symbolic play, and participation. The participation domain includes participation type, cooperation, humor, and language. The Turkish adaptation provides separate forms for children aged 48–59 months and 60–72 months [37]; in the present study, the 48–59-month form was administered to children in the 4-year age group, and the 60–72-month form was administered to children in the 5- and 6-year age groups, based on the child’s age in completed years at assessment. During observation, play behaviors are scored as 1 when the developmentally appropriate play behavior is observed, 0 when the behavior is not observed, and −1 when the child has the opportunity but cannot perform the expected behavior. Item scores are summed across all items to produce an overall score (range −16 to 3 in the present sample), with higher (less negative) scores indicating more consistent performance of developmentally expected play behaviors; this summed score, rather than play age, was used as the play performance variable in the present analyses. Separately, the scale can also be used to derive the child’s play age, and a difference of 8 months or more between chronological age and play age (in either direction) is described in the Turkish adaptation documentation as indicating limitations in play skills [37]; play age itself was not used as an analysis variable in this study. The validity and reliability study of the Turkish version was conducted by Değirmenci [37].

### 2.9. Test of Playfulness

Children’s playfulness was assessed using the Test of Playfulness, Version 4. The scale was developed to evaluate children’s playfulness characteristics during play. It includes four main parameters: perception of control, source of motivation, freedom from reality, and framing. The scale consists of 29 items scored between 0 and 3 under the dimensions of extent, intensity, and skill [17]; the total score used in the present analyses was calculated as the sum of the item-level ratings across all 29 items, yielding a theoretical range of 0–87, with higher scores indicating greater playfulness. The Turkish validity and reliability study of the Test of Playfulness was conducted by Sicim Sevim [38].

### 2.10. Statistical Analysis

Statistical analyses were performed using IBM SPSS Statistics version 22.0. The normality of continuous variables was assessed using the Shapiro–Wilk test. Descriptive data were presented as mean ± standard deviation, median with interquartile range, minimum–maximum values, and frequency with percentage, as appropriate.

As the continuous variables did not meet the normality assumption, non-parametric tests were used. Spearman’s rho correlation analysis was performed to examine the relationships between maternal anxiety, play-related outcomes, and sensory processing scores. The Mann–Whitney U test was used to compare two independent groups, including sex, school type, comorbid ADHD status, maternal education level, and maternal employment status. Because only one mother in the sample had secondary-school education, maternal education level was dichotomized as “high school or below” versus “university” for these group comparisons. The Kruskal–Wallis test was used to compare variables across age groups. Effect sizes for Mann–Whitney U tests were calculated using the formula r = |Z|/√*N* and interpreted as small, moderate, and large for values of 0.10, 0.30, and 0.50, respectively.

Multiple linear regression analysis was performed to identify factors independently associated with maternal anxiety. The Beck Anxiety Inventory score was entered as the dependent variable, while maternal education level, comorbid ADHD status, maternal employment status, school type, and Revised Knox Preschool Play Scale score were entered as independent variables. Regression assumptions were evaluated using standardized residuals, Cook’s distance, leverage values, residual plots, and variance inflation factor values. Because child age and sex were not included in the primary model, sensitivity analyses were performed in which (i) child age (in completed years) and (ii) child age and sex were added to the primary model, and (iii) the primary model was re-estimated after excluding the children recorded as 6 years old (school type was omitted from this last model because it was constant in the remaining sample). A *p*-value of <0.05 was considered statistically significant.

The six correlations between maternal anxiety and children’s developmental play performance, playfulness, and the four main sensory-processing patterns were prespecified as the primary hypothesis-driven analyses; all other analyses were considered secondary/exploratory. To account for multiple comparisons, the Benjamini–Hochberg false discovery rate (FDR) procedure (Q = 0.05) was applied to the two-sided *p*-values separately within each of three families of tests, defined by analysis type, without pooling across families: (1) the 15 pairwise Spearman correlations among the Beck Anxiety Inventory, the Revised Knox Preschool Play Scale, the Test of Playfulness, and the four sensory-processing patterns (the six correlations involving maternal anxiety were the prespecified primary analyses, and the remaining nine were included in the same family); (2) the 42 Spearman correlations of the 14 sensory-processing domains with maternal anxiety, the Revised Knox Preschool Play Scale, and the Test of Playfulness (14 domains × 3 outcomes, treated as one family); and (3) the 35 Mann–Whitney U comparisons of the three outcome measures and the four sensory-processing patterns (seven outcomes) across the five grouping variables (child sex, school type, comorbid ADHD, maternal education, and maternal employment; 5 × 7 comparisons). The regression analyses were not included in any family. Findings are marked with an asterisk in the tables when they remained significant after FDR correction within their family, and are described as exploratory in the text when they did not.

## 3. Results

A total of 58 children diagnosed with ASD aged 4–6 years and their mothers were included in the study. The mean age of the children was 5.12 ± 0.77 years, and the sample included 29 girls (50.0%) and 29 boys (50.0%). Of the children, 41 (70.7%) attended preschool and 17 (29.3%) attended primary school. Comorbid ADHD was present in 10 children (17.2%), and 56 children (96.6%) had a previous therapy history. The mean maternal age was 35.95 ± 3.38 years; 41 mothers (70.7%) were university graduates and 32 (55.2%) were employed (Table 2).

The descriptive statistics of the outcome measures and sensory processing scores are presented in Table 3. The mean Beck Anxiety Inventory score was 5.16 ± 4.00, with a median value of 3.50 (IQR: 2.00–8.00). The mean Revised Knox Preschool Play Scale score was −2.31 ± 4.19, and the mean Test of Playfulness score was 63.91 ± 15.20. Among the sensory processing patterns, the mean scores were 94.74 ± 15.26 for low registration, 129.00 ± 21.53 for sensation-seeking, 80.86 ± 11.22 for sensory sensitivity, and 98.38 ± 16.23 for sensation avoiding.

Spearman correlation analysis showed that the Beck Anxiety Inventory score was moderately and negatively correlated with the Revised Knox Preschool Play Scale score (ρ = −0.564, *p* < 0.001) and the Test of Playfulness score (ρ = −0.492, *p* < 0.001). The Beck Anxiety Inventory score was positively correlated with low registration (ρ = 0.474, *p* < 0.001) and sensation-seeking (ρ = 0.343, *p* = 0.008), whereas it was not significantly correlated with sensory sensitivity or sensation avoiding. These four prespecified, hypothesis-driven correlations remained significant after Benjamini–Hochberg FDR correction. The Revised Knox Preschool Play Scale and the Test of Playfulness were strongly and positively correlated with each other (ρ = 0.953, *p* < 0.001); this unusually high correlation is considered further in the Discussion. Both play-related measures also showed positive correlations with selected sensory processing patterns, particularly sensation avoiding and, for the Test of Playfulness, sensory sensitivity; after FDR correction, the correlations of sensation avoiding with both play measures and of sensory sensitivity with the Test of Playfulness remained significant, whereas the correlation between the Revised Knox Preschool Play Scale and sensory sensitivity did not survive correction and should be interpreted as exploratory (Table 4).

**Table 4:** Correlations between maternal anxiety, play outcomes, and sensory processing patterns.

Reference Variable	Correlated Variable	Spearman’s ρ	*p*	95% CI
Beck Anxiety Inventory	Revised Knox Preschool Play Scale	−0.564	<0.001 *	[−0.718, −0.358]
	Test of Playfulness	−0.492	<0.001 *	[−0.666, −0.268]
	Low registration	0.474	<0.001 *	[0.246, 0.652]
	Sensation seeking	0.343	0.008 *	[0.093, 0.552]
	Sensory sensitivity	0.077	0.566	[−0.185, 0.329]
	Sensation avoiding	−0.050	0.707	[−0.304, 0.211]
Revised Knox Preschool Play Scale	Test of Playfulness	0.953	<0.001	[0.922, 0.972]
	Low registration	0.018	0.893	[−0.241, 0.275]
	Sensation seeking	0.005	0.969	[−0.254, 0.263]
	Sensory sensitivity	0.263	0.046	[0.005, 0.488]
	Sensation avoiding	0.503	<0.001 *	[0.281, 0.674]
Test of Playfulness	Low registration	0.175	0.190	[−0.087, 0.415]
	Sensation seeking	0.162	0.224	[−0.101, 0.403]
	Sensory sensitivity	0.406	0.002 *	[0.165, 0.601]
	Sensation avoiding	0.633	<0.001 *	[0.448, 0.766]

Spearman’s rho correlation analysis was used. ρ: Spearman correlation coefficient; CI: Confidence intervals. *p* < 0.05 was considered statistically significant. 95% confidence intervals were calculated for Spearman’s rho using Fisher’s z transformation. * Remained statistically significant after Benjamini–Hochberg false discovery rate (FDR) correction (Q = 0.05).

Detailed, exploratory correlations between sensory processing domains, maternal anxiety, and play-related outcomes are presented in Table 5. The Beck Anxiety Inventory score showed significant positive correlations with auditory processing (ρ = 0.333, *p* = 0.011), visual processing (ρ = 0.655, *p* < 0.001), oral sensory processing (ρ = 0.426, *p* = 0.001), modulation related to body position and movement (ρ = 0.317, *p* = 0.015), modulation of visual input affecting emotional responses and activity level (ρ = 0.302, *p* = 0.021), and behavioral outcomes of sensory processing (ρ = 0.686, *p* < 0.001). In contrast, the Beck Anxiety Inventory score was negatively correlated with sensory processing related to endurance/tone (ρ = −0.347, *p* = 0.008) and emotional/social responses (ρ = −0.653, *p* < 0.001). All eight of these domain-level correlations with maternal anxiety remained significant after FDR correction; the remaining, non-significant domain-level correlations with maternal anxiety are reported in Table 5 without further comment.

**Table 5:** Correlations of sensory processing domains with maternal anxiety and play-related outcomes.

Sensory Processing Domain	Beck Anxiety Inventory ρ (*p*)	Revised Knox Preschool Play Scale ρ (*p*)	Test of Playfulness ρ (*p*)
Auditory processing	0.333 (0.011) *	0.109 (0.413)	0.263 (0.046)
Visual processing	0.655 (<0.001) *	−0.087 (0.517)	0.054 (0.687)
Vestibular processing	0.184 (0.168)	0.252 (0.056)	0.371 (0.004) *
Touch processing	0.220 (0.097)	0.141 (0.292)	0.286 (0.030)
Multisensory processing	0.188 (0.157)	0.139 (0.299)	0.293 (0.025)
Oral sensory processing	0.426 (0.001) *	−0.018 (0.892)	0.128 (0.339)
Sensory processing related to endurance/tone	−0.347 (0.008) *	0.738 (<0.001) *	0.811 (<0.001) *
Modulation related to body position and movement	0.317 (0.015) *	−0.009 (0.945)	0.145 (0.276)
Modulation of movement affecting activity level	−0.071 (0.595)	0.381 (0.003) *	0.490 (<0.001) *
Modulation of sensory input affecting emotional responses	0.038 (0.777)	0.182 (0.171)	0.328 (0.012) *
Modulation of visual input affecting emotional responses and activity level	0.302 (0.021) *	0.239 (0.070)	0.383 (0.003) *
Emotional/social responses	−0.653 (<0.001) *	0.852 (<0.001) *	0.816 (<0.001) *
Behavioral outcomes of sensory processing	0.686 (<0.001) *	−0.314 (0.016) *	−0.153 (0.253)
Items indicating thresholds for response	−0.053 (0.692)	0.323 (0.013) *	0.396 (0.002) *

Spearman’s rho correlation analysis was used. Values are presented as ρ (*p*). ρ: Spearman correlation coefficient. *p* < 0.05 was considered statistically significant. * Remained statistically significant after Benjamini–Hochberg false discovery rate (FDR) correction (Q = 0.05).

The Revised Knox Preschool Play Scale was positively correlated with sensory processing related to endurance/tone (ρ = 0.738, *p* < 0.001), modulation of movement affecting activity level (ρ = 0.381, *p* = 0.003), emotional/social responses (ρ = 0.852, *p* < 0.001), and items indicating thresholds for response (ρ = 0.323, *p* = 0.013), whereas it was negatively correlated with behavioral outcomes of sensory processing (ρ = −0.314, *p* = 0.016); all five of these domain-level correlations remained significant after FDR correction. The Test of Playfulness was significantly and positively correlated with several sensory processing domains, most robustly with sensory processing related to endurance/tone, modulation of movement affecting activity level, modulation of sensory input affecting emotional responses, modulation of visual input affecting emotional responses and activity level, vestibular processing, emotional/social responses, and items indicating thresholds for response, all of which remained significant after FDR correction. Correlations of the Test of Playfulness with touch processing (ρ = 0.286, *p* = 0.030) and multisensory processing (ρ = 0.293, *p* = 0.025) were nominally significant but did not survive FDR correction and should be interpreted as exploratory (Table 5).

Child-related group comparisons are presented in Table 6. No significant differences were found between girls and boys in the Beck Anxiety Inventory, Revised Knox Preschool Play Scale, or Test of Playfulness scores (*p* > 0.05). Among the four main sensory processing patterns, only low registration differed significantly by sex, with girls showing higher scores than boys (U = 255.000, Z = −2.576, *p* = 0.010, r = 0.34); this difference remained significant after FDR correction.

**Table 6:** Child-related group comparisons of maternal anxiety, play outcomes, and sensory processing patterns.

Group Variable	Outcome	Group 1	Group 2	U	Z	*p*	r
Sex	Beck Anxiety Inventory	Female: 5.62 ± 4.11; 4.00 (2.00–8.50)	Male: 4.69 ± 3.90; 3.00 (2.00–8.00)	353.500	−1.051	0.293	0.14
	Revised Knox Preschool Play Scale	Female: −2.34 ± 4.76; 0.00 (−5.00–1.00)	Male: −2.28 ± 3.61; −2.00 (−4.00–1.00)	399.500	−0.329	0.742	0.04
	Test of Playfulness	Female: 64.52 ± 17.10; 69.00 (58.00–75.50)	Male: 63.31 ± 13.32; 61.00 (54.00–75.50)	375.000	−0.708	0.479	0.09
	Low registration	Female: 98.83 ± 12.13; 96.00 (88.50–109.00)	Male: 90.66 ± 17.10; 88.00 (77.00–95.50)	255.000	−2.576	0.010 *	0.34
	Sensation seeking	Female: 131.00 ± 21.29; 130.00 (115.50–149.50)	Male: 127.00 ± 21.96; 122.00 (110.00–145.00)	364.000	−0.879	0.379	0.12
	Sensory sensitivity	Female: 83.03 ± 10.84; 81.00 (74.50–88.50)	Male: 78.69 ± 11.36; 75.00 (73.00–81.50)	300.500	−1.869	0.062	0.25
	Sensation avoiding	Female: 101.21 ± 14.59; 103.00 (89.50–111.50)	Male: 95.55 ± 17.52; 93.00 (79.00–110.50)	339.000	−1.268	0.205	0.17
School type	Beck Anxiety Inventory	Preschool: 6.15 ± 4.28; 6.00 (2.00–9.50)	Primary school: 2.76 ± 1.64; 3.00 (2.00–3.00)	200.000	−2.558	0.011 *	0.34
	Revised Knox Preschool Play Scale	Preschool: −3.17 ± 4.52; −3.00 (−5.50–0.50)	Primary school: −0.24 ± 2.22; 0.00 (−2.00–1.00)	210.500	−2.372	0.018	0.31
	Test of Playfulness	Preschool: 60.88 ± 16.18; 61.00 (52.00–75.00)	Primary school: 71.24 ± 9.40; 72.00 (63.50–78.00)	219.000	−2.213	0.027	0.29
	Low registration	Preschool: 96.63 ± 15.28; 95.00 (86.00–104.00)	Primary school: 90.18 ± 14.63; 88.00 (80.50–95.50)	247.000	−1.735	0.083	0.23
	Sensation seeking	Preschool: 131.02 ± 21.06; 130.00 (114.00–149.00)	Primary school: 124.12 ± 22.52; 118.00 (111.00–137.50)	280.500	−1.162	0.245	0.15
	Sensory sensitivity	Preschool: 80.54 ± 11.24; 79.00 (74.00–85.50)	Primary school: 81.65 ± 11.47; 77.00 (73.50–89.50)	341.500	−0.120	0.905	0.02
	Sensation avoiding	Preschool: 97.98 ± 16.52; 97.00 (82.50–109.50)	Primary school: 99.35 ± 15.98; 99.00 (84.00–114.50)	327.500	−0.359	0.720	0.05
Comorbid ADHD	Beck Anxiety Inventory	Yes: 10.10 ± 2.77; 9.50 (7.75–13.00)	No: 4.13 ± 3.41; 3.00 (2.00–5.75)	48.000	−3.985	<0.001 *	0.52
	Revised Knox Preschool Play Scale	Yes: −7.50 ± 5.21; −6.00 (−11.75–−4.75)	No: −1.23 ± 3.02; 0.00 (−3.00–1.00)	67.000	−3.583	<0.001 *	0.47
	Test of Playfulness	Yes: 45.10 ± 16.86; 42.50 (35.25–55.25)	No: 67.83 ± 11.61; 70.50 (58.25–76.00)	67.500	−3.553	<0.001 *	0.47
	Low registration	Yes: 102.10 ± 15.35; 98.50 (90.25–113.25)	No: 93.21 ± 14.94; 91.00 (84.25–99.50)	153.000	−1.792	0.073	0.24
	Sensation seeking	Yes: 141.20 ± 24.17; 149.50 (126.00–153.25)	No: 126.46 ± 20.30; 122.50 (112.25–137.75)	147.500	−1.904	0.057	0.25
	Sensory sensitivity	Yes: 78.80 ± 13.59; 75.50 (69.25–86.25)	No: 81.29 ± 10.78; 79.00 (74.00–86.75)	193.000	−0.969	0.333	0.13
	Sensation avoiding	Yes: 89.90 ± 19.67; 79.50 (76.25–109.50)	No: 100.15 ± 15.07; 101.00 (88.25–110.75)	148.500	−1.884	0.060	0.25

Group values are presented as mean ± standard deviation; median (Q1–Q3). Mann–Whitney U test was used. Effect size was calculated as r = |Z|/√N. ADHD: attention-deficit/hyperactivity disorder. *p* < 0.05 was considered statistically significant. * Remained statistically significant after Benjamini–Hochberg false discovery rate (FDR) correction (Q = 0.05).

School type was associated with maternal anxiety and play-related outcomes in univariate comparisons. Children attending preschool had higher Beck Anxiety Inventory scores than children attending primary school (U = 200.000, Z = −2.558, *p* = 0.011, r = 0.34); this difference remained significant after FDR correction. In contrast, children attending primary school had higher Revised Knox Preschool Play Scale scores (U = 210.500, Z = −2.372, *p* = 0.018, r = 0.31) and Test of Playfulness scores (U = 219.000, Z = −2.213, *p* = 0.027, r = 0.29) than those attending preschool; neither of these differences survived FDR correction, and both should be interpreted as exploratory. No significant differences were found between school type groups in the four main sensory processing patterns (*p* > 0.05).

Children with comorbid ADHD had significantly higher Beck Anxiety Inventory scores than those without comorbid ADHD (U = 48.000, Z = −3.985, *p* < 0.001, r = 0.52). In addition, children with comorbid ADHD had significantly lower Revised Knox Preschool Play Scale scores (U = 67.000, Z = −3.583, *p* < 0.001, r = 0.47) and Test of Playfulness scores (U = 67.500, Z = −3.553, *p* < 0.001, r = 0.47); all three of these differences remained significant after FDR correction, notwithstanding the small size of the ASD + ADHD subgroup (*n* = 10). No significant differences were found in the four main sensory processing patterns according to comorbid ADHD status (*p* > 0.05).

Age-group comparisons were performed using the Kruskal–Wallis test. No significant differences were found among age groups in maternal anxiety, play-related outcomes, or sensory processing pattern scores (*p* > 0.05 for all).

Maternal-related group comparisons are presented in Table 7. Maternal education level was not associated with significant differences in Beck Anxiety Inventory, Revised Knox Preschool Play Scale, or Test of Playfulness scores (*p* > 0.05). Among the sensory processing patterns, sensation-seeking and sensory sensitivity differed significantly according to maternal education level. Children of university-educated mothers had higher sensation-seeking scores (U = 161.000, Z = −3.204, *p* = 0.001, r = 0.42), a difference that remained significant after FDR correction, and higher sensory sensitivity scores (U = 222.500, Z = −2.155, *p* = 0.031, r = 0.28), a difference that did not survive FDR correction and should be interpreted as exploratory.

Maternal employment status was significantly associated with maternal anxiety and play-related outcomes. Unemployed mothers had higher Beck Anxiety Inventory scores than employed mothers (U = 243.500, Z = −2.719, *p* = 0.007, r = 0.36); children of employed mothers had higher Revised Knox Preschool Play Scale scores (U = 216.000, Z = −3.146, *p* = 0.002, r = 0.41) and Test of Playfulness scores (U = 213.000, Z = −3.176, *p* = 0.001, r = 0.42) than children of unemployed mothers; all three of these differences remained significant after FDR correction. Among sensory processing patterns, sensation avoiding was nominally higher in children of employed mothers than in children of unemployed mothers (U = 268.500, Z = −2.307, *p* = 0.021, r = 0.30), but this difference did not survive FDR correction and should be interpreted as exploratory.

**Table 7:** Maternal-related group comparisons of maternal anxiety, play outcomes, and sensory processing patterns.

Group Variable	Outcome	Group 1	Group 2	U	Z	*p*	r
Maternal education	Beck Anxiety Inventory	High school or below: 5.59 ± 4.05; 3.00 (2.00–9.50)	University: 4.98 ± 4.02; 4.00 (2.00–8.00)	307.000	−0.715	0.475	0.09
	Revised Knox Preschool Play Scale	High school or below: −3.59 ± 5.61; −2.00 (−7.50–0.50)	University: −1.78 ± 3.37; −1.00 (−4.00–1.00)	300.000	−0.834	0.404	0.11
	Test of Playfulness	High school or below: 59.12 ± 19.63; 68.00 (40.50–75.00)	University: 65.90 ± 12.70; 68.00 (56.50–76.00)	289.500	−1.008	0.313	0.13
	Low registration	High school or below: 91.71 ± 13.46; 89.00 (84.50–99.50)	University: 96.00 ± 15.93; 94.00 (85.00–107.00)	294.000	−0.932	0.352	0.12
	Sensation seeking	High school or below: 115.41 ± 19.20; 113.00 (104.00–122.50)	University: 134.63 ± 20.05; 131.00 (119.50–150.00)	161.000	−3.204	0.001 *	0.42
	Sensory sensitivity	High school or below: 76.12 ± 9.53; 75.00 (73.00–79.00)	University: 82.83 ± 11.38; 80.00 (74.00–88.50)	222.500	−2.155	0.031	0.28
	Sensation avoiding	High school or below: 93.41 ± 16.54; 89.00 (77.50–110.00)	University: 100.44 ± 15.85; 102.00 (86.50–112.50)	263.000	−1.461	0.144	0.19
Maternal employment	Beck Anxiety Inventory	Employed: 4.00 ± 3.69; 2.50 (2.00–5.50)	Unemployed: 6.58 ± 3.97; 6.00 (3.00–10.25)	243.500	−2.719	0.007 *	0.36
	Revised Knox Preschool Play Scale	Employed: −0.91 ± 3.35; 0.50 (−3.75–1.75)	Unemployed: −4.04 ± 4.51; −3.00 (−6.00–−0.75)	216.000	−3.146	0.002 *	0.41
	Test of Playfulness	Employed: 69.47 ± 12.13; 73.00 (60.25–78.00)	Unemployed: 57.08 ± 16.01; 58.50 (41.75–70.25)	213.000	−3.176	0.001 *	0.42
	Low registration	Employed: 95.88 ± 14.56; 94.00 (85.25–103.25)	Unemployed: 93.35 ± 16.25; 92.00 (82.25–100.25)	373.000	−0.673	0.501	0.09
	Sensation seeking	Employed: 131.84 ± 18.43; 127.50 (118.25–144.75)	Unemployed: 125.50 ± 24.76; 119.00 (104.50–149.25)	333.000	−1.298	0.194	0.17
	Sensory sensitivity	Employed: 82.03 ± 11.28; 79.50 (74.00–87.75)	Unemployed: 79.42 ± 11.20; 75.00 (73.75–82.25)	344.000	−1.127	0.260	0.15
	Sensation avoiding	Employed: 102.50 ± 14.21; 105.00 (91.50–113.25)	Unemployed: 93.31 ± 17.38; 89.00 (77.75–108.25)	268.500	−2.307	0.021	0.30

Group values are presented as mean ± standard deviation; median (Q1–Q3). Mann–Whitney U test was used. Effect size was calculated as r = |Z|/√N. *p* < 0.05 was considered statistically significant. * Remained statistically significant after Benjamini–Hochberg false discovery rate (FDR) correction (Q = 0.05).

The results of the multiple linear regression analysis are presented in Table 8. The five candidate predictors—maternal education, maternal employment, school type, comorbid ADHD, and developmental play performance—were selected to represent the primary sociodemographic (maternal education, employment, school type) and clinical/functional (ADHD, play performance) domains of interest. Sensory-processing variables were not entered into the multivariable model given their conceptual and empirical overlap with the play measures (Table 4 and Table 5), which could introduce collinearity; child age and sex were not included given the absence of significant univariate associations with maternal anxiety in this sample (Table 6). Given these considerations, the regression model is best regarded as descriptive/exploratory rather than as a confirmatory test of a fully prespecified model. Multiple linear regression analysis was performed to identify factors independently associated with maternal anxiety. Maternal education level, comorbid ADHD, maternal employment status, school type, and Revised Knox Preschool Play Scale score were included in the model. The regression model was statistically significant and explained 51.6% of the variance in Beck Anxiety Inventory scores (R^2^ = 0.516, adjusted R^2^ = 0.469, F(5,52) = 11.066, *p* < 0.001).

**Table 8:** Linear regression analysis for predictors of maternal anxiety.

Predictor	B	SE	β	t	*p*	95% CI for B	VIF
Maternal education level	0.787	0.968	0.090	0.813	0.420	−1.156 to 2.730	1.326
Comorbid ADHD	2.871	1.262	0.274	2.276	0.027	0.340 to 5.403	1.551
Maternal employment	−1.072	0.951	−0.134	−1.128	0.265	−2.980 to 0.836	1.526
School type	−1.269	0.916	−0.146	−1.386	0.172	−3.106 to 0.568	1.186
Revised Knox Preschool Play Scale	−0.393	0.122	−0.411	−3.226	0.002	−0.637 to −0.149	1.745

Dependent variable: Beck Anxiety Inventory score. B: unstandardized coefficient; SE: standard error; β: standardized coefficient; CI: confidence interval; VIF: variance inflation factor; ADHD: attention-deficit/hyperactivity disorder. Model fit: R^2^ = 0.516, adjusted R^2^ = 0.469, F(5,52) = 11.066, *p* < 0.001. Categorical predictors were coded as follows: maternal education (university = 1, high school or below = 0), comorbid ADHD (yes = 1, no = 0), maternal employment (employed = 1, unemployed = 0), and school type (primary school = 1, preschool = 0).

Comorbid ADHD was significantly and positively associated with maternal anxiety in the multivariable model (B = 2.871, β = 0.274, *p* = 0.027). The Revised Knox Preschool Play Scale score was significantly and negatively associated with maternal anxiety (B = −0.393, β = −0.411, *p* = 0.002). Maternal education level, maternal employment status, and school type were not significantly associated with maternal anxiety in this model (*p* > 0.05). VIF values ranged from 1.186 to 1.745, indicating no multicollinearity problem. Sensitivity analyses are presented in Table 9. The association of the Revised Knox Preschool Play Scale score with maternal anxiety was unchanged when child age or child age and sex were added to the model, and the association of comorbid ADHD was also preserved; neither child age nor sex was independently associated with maternal anxiety. Child age and school type were strongly related in this sample (all 17 children attending primary school were 6 years old, and none of the 37 children aged 4–5 years attended primary school), and the school type coefficient was attenuated when age was added. When the 21 children recorded as 6 years old were excluded (*n* = 37; school type omitted because it was constant), the association of the Knox score remained significant (β = −0.560, *p* < 0.001), whereas that of comorbid ADHD retained a similar magnitude but was imprecisely estimated (B = 2.467, 95% CI −0.655 to 5.590, *p* = 0.117; B = 3.267, *p* = 0.043 when sex was also included), reflecting the small number of children with ADHD in this subsample (*n* = 7).

**Table 9:** Sensitivity analyses of the multiple linear regression model for maternal anxiety (Beck Anxiety Inventory score).

Predictor	Model 1 Primary Model (n = 58)	Model 2 + Child Age (n = 58)	Model 3 + Child Age and Sex (n = 58)	Model 4 Children Aged 4–5 Years (n = 37)	Model 5 Children Aged 4–5 Years + Sex (n = 37)
Maternal education level	0.787 (−1.156 to 2.730) *p* = 0.420	0.638 (−1.390 to 2.667) *p* = 0.530	0.541 (−1.484 to 2.566) *p* = 0.594	0.114 (−2.590 to 2.819) *p* = 0.932	−0.523 (−3.236 to 2.190) *p* = 0.697
Comorbid ADHD	2.871 (0.340 to 5.403) *p* = 0.027	2.914 (0.360 to 5.469) *p* = 0.026	3.108 (0.546 to 5.669) *p* = 0.018	2.467 (−0.655 to 5.590) *p* = 0.117	3.267 (0.115 to 6.420) *p* = 0.043
Maternal employment	−1.072 (−2.980 to 0.836) *p* = 0.265	−1.060 (−2.982 to 0.862) *p* = 0.273	−0.958 (−2.878 to 0.962) *p* = 0.321	−0.231 (−2.693 to 2.232) *p* = 0.850	0.071 (−2.336 to 2.477) *p* = 0.953
School type	−1.269 (−3.106 to 0.568) *p* = 0.172	−0.677 (−3.504 to 2.151) *p* = 0.633	−0.685 (−3.499 to 2.129) *p* = 0.627	–	–
Revised Knox Preschool Play Scale	−0.393 (−0.637 to −0.149) *p* = 0.002	−0.401 (−0.649 to −0.153) *p* = 0.002	−0.393 (−0.640 to −0.146) *p* = 0.002	−0.623 (−0.960 to −0.286) *p* < 0.001	−0.606 (−0.933 to −0.279) *p* < 0.001
Child age (years)	–	−0.442 (−2.038 to 1.154) *p* = 0.580	−0.403 (−1.992 to 1.187) *p* = 0.613	–	–
Child sex	–	–	0.968 (−0.594 to 2.530) *p* = 0.219	–	1.946 (−0.244 to 4.137) *p* = 0.080
R^2^ (adjusted R^2^)	0.516 (0.469)	0.518 (0.462)	0.533 (0.468)	0.487 (0.422)	0.536 (0.461)

Values are unstandardized coefficients (B) with 95% confidence intervals in parentheses and *p*-values; statistically significant coefficients (*p* < 0.05) are shown in bold. Dependent variable: Beck Anxiety Inventory score. Coding: maternal education (university = 1, high school or below = 0), comorbid ADHD (yes = 1, no = 0), maternal employment (employed = 1, unemployed = 0), school type (primary school = 1, preschool = 0), child sex (female = 1, male = 0); child age was entered as completed years. School type was omitted from Models 4 and 5 because all children aged 4–5 years attended preschool. ADHD: attention-deficit/hyperactivity disorder.

Regression assumptions were evaluated using standardized residuals, Cook’s distance, leverage values, and residual plots. The standardized residuals ranged from −1.62 to 3.14, and the maximum Cook’s distance value was 0.175, indicating no influential observations. Visual inspection of the P–P plot and scatterplot suggested that the assumptions of residual normality and homoscedasticity were acceptable.

## 4. Discussion

This study examined, in 58 mother–child dyads of children aged 4–6 years with autism spectrum disorder, the relationships between maternal anxiety and children’s developmental play performance, playfulness, and sensory-processing characteristics, along with the factors independently associated with maternal anxiety. Higher maternal anxiety was moderately associated with poorer developmental play performance and lower playfulness, and was further related to specific sensory-processing patterns—positively to low registration and sensation-seeking raw scores, indicating that higher maternal anxiety was associated with more typical (i.e., less atypical) processing in these two specific patterns rather than with more pronounced sensory difficulty—but not to sensory sensitivity or sensation avoiding. Play outcomes, in turn, were most consistently related to sensory sensitivity, sensation avoiding, and several sensory-emotional subdomains. In a multivariable model, developmental play performance was independently and negatively associated with maternal anxiety; comorbid attention-deficit/hyperactivity disorder (ADHD) was also positively associated with maternal anxiety, but this finding is preliminary because it is based on only 10 children with ADHD, and the model as a whole explained 51.6% of the variance in maternal anxiety scores; several sociodemographic group differences observed in univariate comparisons—maternal education, employment status, and school type—did not retain independent significance once these clinical variables were accounted for. Overall, the findings suggest that maternal anxiety in this population was most strongly associated with the child’s play performance, with a preliminary association with co-occurring ADHD and a more limited independent contribution from sociodemographic factors; because the design is cross-sectional, these associations should not be read as evidence of temporal or causal prediction.

Maternal anxiety was moderately and negatively correlated with both developmental play performance (ρ = −0.564) and playfulness (ρ = −0.492), and developmental play performance remained independently and negatively associated with maternal anxiety after other child- and family-level variables were accounted for. This cross-sectional pattern is consistent with the possibility of a reciprocal, transactional relationship between maternal anxiety and children’s play, but it should not be read as evidence that maternal anxiety causes poorer play, or the reverse. Comparable direct evidence linking maternal anxiety specifically to play is scarce: the closest available evidence comes from a small longitudinal study of non-autistic mother–toddler dyads, in which higher maternal anxiety was associated with less maternal and child pretend play [39], although that sample was not autistic and the outcome was concurrent rather than predictive of later play. Within autism-specific research, developmental capacity itself is a strong competing explanation for the present correlation: pretend-play ability has been shown to relate to playfulness even after accounting for autism symptom severity, verbal comprehension, and age [40], and autistic preschoolers show lower play and playfulness scores than typically developing peers, with ideational praxis predicting both [41]. It is therefore plausible that children’s play difficulties reflect broader developmental characteristics that also elevate maternal anxiety, rather than anxiety directly impairing children’s play, although the present cross-sectional design cannot adjudicate between these accounts.

In this small subgroup (*n* = 10), comorbid ADHD was associated with higher maternal anxiety and with lower scores on both play measures, and the association with maternal anxiety remained in the multivariable model after developmental play performance, school type, and maternal sociodemographic variables were taken into account; these findings are preliminary. This is consistent with prior reports that mothers of children with co-occurring ASD and ADHD experience greater parenting stress than mothers of children with ASD alone, and that ADHD symptoms are independently associated with poorer adaptive and social functioning in autistic children [42,43]. Inattention in particular has been linked to social impairment in children with both diagnoses [44], which may plausibly increase the amount of adult prompting, supervision, and support required during play and daily routines, although this specific caregiving link was not directly tested in that study. Prior studies have generally not tested whether ADHD comorbidity predicts maternal anxiety independently of overlapping child- and family-level factors [45,46]; the present model suggests that, in this sample, ADHD comorbidity retained an association with maternal anxiety after adjusting for developmental play performance and sociodemographic variables, although the estimate was imprecise when the analysis was restricted to children aged 4–5 years (Table 9). Because the cross-sectional design cannot establish directionality, because the ASD + ADHD subgroup comprised only 10 children, and because the procedures underlying the pre-existing ADHD diagnoses could not be verified (see Limitations), this finding should be regarded as preliminary and confirmed in larger, prospective samples with multi-informant measures of child play, caregiving demands, and parental wellbeing.

Maternal anxiety was moderately and positively correlated with low registration (ρ = 0.474) and, more weakly, with sensation-seeking (ρ = 0.343), but showed no significant association with sensory sensitivity or sensation avoiding. Because higher scores on these patterns indicate less frequent occurrence of the corresponding atypical sensory behavior, these associations indicate that lower maternal anxiety was linked to more pronounced low-registration and sensation-seeking difficulties in the child, whereas higher maternal anxiety was linked to more typical processing in these two patterns specifically. This direction was unexpected. Previous studies of children with ASD have generally reported that more pronounced sensory features are associated with poorer maternal outcomes: in one study, all four sensory quadrants, including low registration and seeking, were positively associated with caregiver strain, with sensory avoiding showing the strongest association [47], and in a study of toddlers with ASD, parent-reported sensory reactivity was correlated with maternal anxiety and depressive symptoms [48]. We are not aware of previous studies reporting that more typical low-registration and sensation-seeking scores were associated with higher maternal anxiety, so the present finding is not supported by earlier literature and should be considered exploratory. Several explanations, none of which could be tested here, may be considered. First, because both measures were maternal-report, the association may partly reflect response style. However, the reporting-bias literature has more often found that mothers with higher anxiety or depressive symptoms report more rather than fewer child problems, including symptoms not corroborated by other informants [49], in preschoolers with ASD, maternal depression was associated with questionnaire-based ratings of ASD behaviors more strongly than with interview or observational measures [50], and in autistic children aged 2–6 years, higher parenting stress was associated with more negative parent ratings of problem behavior and social impairment, whereas blinded clinician ratings did not differ [51]. Shared-informant effects would therefore be expected to inflate associations in the expected direction rather than to produce more typical ratings, and the low and restricted range of anxiety scores in this sample makes a strong reporting distortion less likely; this explanation alone is therefore unlikely to account for the direction observed. Second, more anxious mothers may adapt the child’s environment and daily activities more closely, or compensate for missed responses through repeated prompting, which could reduce the opportunity for, or the visibility of, seeking and low-registration behaviors in maternal ratings. Parental anxiety has been associated with family accommodation of anxiety in families of autistic children [52], and families of children with sensory over-responsivity report accommodating sensory challenges in daily routines [53]. Maternal stress may also influence how child behavior is rated, although not uniformly in one direction: a conference abstract reported that caregiver stress moderated the agreement between caregiver-reported and clinician-observed sensation-seeking scores, although the direction of this effect was not specified [54]. However, whether accommodation or compensation of this kind reduces sensation-seeking behavior, or its visibility in maternal ratings, has not been tested; exploratory behavior should not be equated with sensation-seeking; this account applies less directly to low registration; and fewer reported missed responses should not be taken to indicate better sensory registration. Third, the cross-sectional design cannot establish the direction of influence. Longitudinal work in toddlers with ASD indicates that early sensory over-responsivity predicted later family impairment and parenting stress independently of autism severity, child emotional problems, and maternal anxiety and depressive symptoms [55], so a child-to-mother pathway cannot be excluded, although it would not by itself explain why more typical scores accompany higher anxiety. Fourth, an unmeasured third factor, such as the child’s cognitive or language level, autism symptom severity, or ADHD status, may influence both measures. Finally, the correlations were modest (ρ = 0.343–0.474) and were not mirrored for sensory sensitivity or sensation avoiding, the patterns that prior work links more consistently with caregiver strain, which further supports caution in interpreting this result. This pattern-specific profile indicates that maternal anxiety is not uniformly related to all sensory-processing patterns and should not be summarized as a general statement that greater sensory difficulty is associated with greater maternal anxiety; it should be regarded as an exploratory finding warranting replication before any caregiving-demand mechanism is inferred. Population- and pattern-specific data linking maternal anxiety directly to these four sensory-processing patterns in ASD are limited; available caregiver-distress studies more often report on parenting stress broadly or on different pattern groupings, such as an association between an overall avoider pattern and maternal stress [56]. The absence of a significant association with sensory sensitivity or sensation avoiding in the present sample may reflect differences in sample characteristics, context, or measurement approach rather than a true absence of any relationship, and this pattern-specific profile should be examined further before being treated as a stable finding. It should also be noted that both maternal anxiety and children’s sensory-processing scores were derived from maternal reports, whereas the play measures were scored from independent observation; the associations between maternal anxiety and sensory-processing patterns reported here may therefore partly reflect shared-informant or perception effects rather than a purely child-level relationship, an alternative explanation that should be considered alongside the other explanations considered above. The play observation was conducted by a different evaluator than the one who collected the mother-reported measures, which reduces the likelihood that expectancy related to maternal anxiety directly influenced play scoring; however, because the Sensory Profile and Beck Anxiety Inventory were both completed by the same informant (the mother), the shared-informant explanation for associations between these two measures remains applicable.

Beyond the four main patterns, maternal anxiety showed its strongest domain-level associations with behavioral outcomes of sensory processing (ρ = 0.686), visual processing (ρ = 0.655), and emotional/social responses (ρ = −0.653), with more modest associations for auditory processing, oral sensory processing, and modulation related to body position and movement (ρs = 0.30–0.43). Because higher domain-level scores indicate less frequent occurrence of the described difficulty (i.e., more typical processing), these three associations do not share a common interpretive direction and should not be summarized under a single explanation. The negative association with emotional/social responses indicates that higher maternal anxiety was associated with more frequent difficulties in this domain, consistent with a caregiving-burden account in which more atypical emotional/social responses may increase maternal anxiety. By contrast, the positive associations with behavioral outcomes of sensory processing and visual processing indicate that higher maternal anxiety was associated with less frequent difficulties (more typical processing) in these domains-a pattern that runs counter to a straightforward caregiving-burden account and instead mirrors the unexpected direction already noted above for low registration and sensation-seeking. Because the direction of scoring and the precise behavioral referents of these subdomains vary across instruments and studies, and because comparable domain-level data linking maternal anxiety to these specific subdomains in ASD are not well established in the existing literature, these associations are best interpreted as descriptive of this sample rather than as confirmed general patterns.

Both play measures were associated with sensory sensitivity and sensation avoiding (Revised Knox Preschool Play Scale: ρ = 0.263 and 0.503; Test of Playfulness: ρ = 0.406 and 0.633, respectively) and, at the subdomain level, with processing related to endurance/tone, modulation of movement affecting activity level, emotional/social responses, and thresholds for response; playfulness was additionally associated with vestibular, tactile, and multisensory processing. These associations suggest that sensory-motor regulation—particularly the capacity to sustain physical engagement, regulate arousal during movement, and tolerate varied sensory input—may be relevant to how autistic children orient to, explore, and remain engaged with play materials and partners. This is broadly consistent with occupational therapy accounts proposing that modulation-related sensory processing shapes playfulness [57] and that specific sensory patterns differentially relate to developmental participation outcomes [58], although the present cross-sectional design cannot establish that improving sensory modulation would directly improve play outcomes.

The Revised Knox Preschool Play Scale and the Test of Playfulness were very strongly correlated in this sample (ρ = 0.953), despite reflecting conceptually distinct constructs—developmental play performance versus the quality and motivation of engagement, respectively. Such a strong association may partly reflect genuine convergence, as both instruments were scored by the same rater from a single, continuous play observation, and constructs such as intrinsic motivation, initiation, and social engagement plausibly overlap with the organization and content of play captured by the Knox scale. Comparably high convergence has been reported between other observational playfulness and play-skill measures assessed within the same session—for example, between the Test of Playfulness and the Children’s Playfulness Scale [18]—consistent with shared behavioral indicators and shared-method variance when two observational measures are scored from the same play episode, rather than necessarily reflecting fully independent constructs; direct evidence correlating the Revised Knox Preschool Play Scale and the Test of Playfulness specifically could not be identified in the literature reviewed for this study. Because a correlation of this magnitude also raises the possibility of measurement redundancy, both scores were not entered together as independent variables in the same regression model in the present study, and this very strong association should be treated as a notable methodological finding meriting replication with independent raters or occasions, rather than solely as evidence of validity.

Several sociodemographic and clinical group differences emerged in univariate comparisons but call for cautious interpretation. Children attending preschool had mothers with higher anxiety and had lower play scores than children attending primary school, but school type did not retain independent significance in the multivariable model; this raw difference may partly reflect age, developmental expectations, or educational support rather than school type itself, particularly as no differences emerged across child age groups. Child age was not entered as a candidate regression predictor because preliminary Kruskal–Wallis comparisons showed no significant association between age group and maternal anxiety, whereas school type was retained given its significant univariate association with maternal anxiety; because age and school type were in fact strongly related in this sample (all children attending primary school were 6 years old), school type may partly act as a proxy for age. This variable-selection strategy was based on univariate results from the present dataset and is therefore exploratory; to examine its influence, sensitivity analyses adding child age and sex to the model were performed, and these did not materially change the associations of play performance and comorbid ADHD with maternal anxiety (see Results). Future studies should examine age and school type jointly in larger samples to disentangle their relative contributions. Unemployed mothers reported higher anxiety, and their children scored lower on both play measures than employed mothers, yet maternal employment status also did not remain significant once ADHD status and play performance were accounted for; employment status may be entangled with economic resources, social support, caregiving-sharing arrangements, and child functioning rather than exerting an independent effect, and a causal interpretation such as “employment reduces anxiety” is not supported by these data. Maternal education level was not associated with maternal anxiety or play outcomes, though it was associated with two sensory-processing patterns (sensation-seeking and sensory sensitivity); given the number of comparisons performed, this should be treated as an exploratory finding. Child sex and age showed no differences in maternal anxiety or the two play measures; only low registration differed by sex (higher in girls), and sex and age were not included as candidate predictors in the regression model, so this finding cannot be described as having “lost” independent significance in the way that maternal education, employment, and school type did after being tested and found non-significant in the multivariable model. Taken together, sociodemographic differences appeared meaningful in isolation but contributed limited independent information once the child’s play performance and ADHD status were considered.

These findings suggest that occupational therapy assessment of young autistic children should not focus on sensory characteristics or child-level play performance alone, but should also consider maternal anxiety and the broader family routine within which play occurs. Mothers of children with lower play performance, in particular, may benefit from screening for anxiety symptoms as part of a family-centered evaluation; because Beck Anxiety Inventory scores in this sample were low and restricted in range, this recommendation should be understood as identifying mothers who may warrant closer attention or further evaluation, rather than as evidence that the present findings generalize to mothers with clinically significant or diagnosed anxiety. A comprehensive occupational therapy assessment might therefore integrate the child’s sensory-processing profile, developmental play performance and playfulness, daily routines, parent–child interaction, and caregiver wellbeing, rather than treating these as separate concerns. Correspondingly, intervention need not be limited to child-directed sensory strategies; supporting mothers in structuring the play environment, recognizing the child’s sensory needs, engaging in shared play, grading activity demands, and managing challenging behavior and daily routines may be equally relevant, with referral to psychology, child psychiatry, or special education services where indicated. Elevated maternal anxiety identified through such screening should be treated as a marker warranting further evaluation and support, not as a clinical diagnosis in itself. The ADHD-related finding is preliminary and is not used here to support specific clinical recommendations.

This study has several strengths. It evaluated child- and mother-related variables within the same family unit rather than considering them in isolation, and it assessed play using two conceptually distinct, standardized observational instruments capturing complementary aspects of play—developmental performance and playfulness—rather than a single measure, although, as noted in the Limitations, both instruments were scored from the same observation session and were not methodologically independent measurements. Sensory processing was examined at both the level of the four broad patterns and their more specific subdomains; effect sizes were reported alongside *p*-values for all group comparisons, and associations were tested using multivariable regression—including an assessment of model assumptions and multicollinearity—rather than relying solely on pairwise comparisons. The achieved sample of 58 mother–child dyads met the minimum sample size calculated through an a priori power analysis, although it did not reach the slightly larger target set to allow for anticipated data loss, a point discussed further below.

Several limitations should be considered when interpreting these findings. The cross-sectional design precludes any conclusion about the direction or causal nature of the relationships observed between maternal anxiety and children’s characteristics. The study was conducted at a single center with a relatively small sample, which may limit generalizability to other clinical, cultural, and socioeconomic contexts. Because child age was recorded only in completed years rather than in exact months, the precise chronological age in months of each child could not be determined. Because exact age in months was not recorded, it cannot be confirmed that every child assessed with the 60–72-month form of the Turkish adaptation fell within its validated range; in particular, the 21 children recorded as 6 years old could have been up to 83 months old, which would exceed this form’s upper validated bound of 72 months. Exact ages in months could not be retrieved from the clinical records or original assessment documentation. To examine whether this issue could have influenced the findings, we repeated the regression analysis in the 37 children aged 4 and 5 years, all of whom fell within the validated age range of the form they were administered (school type was omitted from this model because all of these children attended preschool); the association between Revised Knox Preschool Play Scale scores and maternal anxiety was preserved and, if anything, stronger (β = −0.56, *p* < 0.001), whereas the association with comorbid ADHD retained a similar magnitude (B = 2.47, 95% CI −0.66 to 5.59) but was imprecisely estimated (*p* = 0.117), reflecting the small number of children with ADHD in this subsample (*n* = 7). Future studies should record and report exact chronological age in months to allow this to be verified. Nonetheless, by jointly examining maternal anxiety alongside children’s sensory processing and play performance—a combination rarely studied together in early childhood ASD—the present findings offer insight of relevance to family-centered occupational therapy practice beyond this specific setting, and future multicenter studies are needed to confirm their generalizability. Overall anxiety levels in this sample were low and had a restricted range (Beck Anxiety Inventory mean = 5.16, range = 1–15), which may have attenuated some associations and should be considered when generalizing these findings to samples with more severe maternal anxiety. The ASD + ADHD subgroup was small (*n* = 10), so subgroup comparisons and the corresponding regression finding require replication in independent, larger samples. Although Benjamini–Hochberg FDR correction was applied within each prespecified family of secondary and exploratory analyses, these subdomain-level and sociodemographic findings remain exploratory in nature; surviving FDR correction reduces, but does not eliminate, the risk of false-positive findings, and does not transform these analyses into confirmatory ones. In addition, the modest sample size limits the precision and stability of individual associations, and all such findings should be interpreted with appropriate caution and replicated in larger, independent samples. Several child-related measures were obtained through maternal reports, introducing the possibility of shared-informant and perception bias. In addition, the Turkish adaptation of the Sensory Profile was validated in children with autism aged 5–10 years, whereas 14 of the present children (24%) were 4 years old, and its authors noted that some items may be less informative in very young Turkish children [36]. That study also reported poor internal consistency in the autism group for subsections I, J, and N (Cronbach’s α = 0.45, 0.27, and 0.28, respectively) and did not report psychometric properties for the four pattern scores [36]; domain-level findings involving these three subsections (modulation of movement affecting activity level, modulation of sensory input affecting emotional responses, and items indicating thresholds for response) should therefore be interpreted with particular caution. In an exploratory (uncorrected) analysis restricted to the 44 children aged 5–6 years, maternal anxiety remained positively correlated with low registration (ρ = 0.38, *p* = 0.011) and sensation-seeking (ρ = 0.31, *p* = 0.039) and negatively correlated with the Revised Knox Preschool Play Scale (ρ = −0.54, *p* < 0.001) and the Test of Playfulness (ρ = −0.42, *p* = 0.005), which suggests that the main findings were not driven by the 4-year-old children. The two play measures were derived from the same observational context, which may have contributed to their very strong intercorrelation and points to a need for future studies using independent raters or occasions. Because a single rater—a recently graduated occupational therapist—scored the Knox and Test of Playfulness assessments, and because sessions were not video- or audio-recorded, no second rater could independently double-code any sessions, and inter-rater or intra-rater reliability for these observational measures could not be assessed in this study; the rater’s relatively limited clinical experience is a further limitation relevant to the reproducibility of the play-observation scores. This should be addressed in future work using independent or repeated coding, ideally with recorded sessions and raters of varying experience levels. Almost all children (96.6%) had a previous therapy history, but the type, duration, and intensity of this prior therapy were not recorded in detail, and this heterogeneity could not be examined as a potential source of variability in children’s play and sensory outcomes. The Turkish adaptation of the Revised Knox Preschool Play Scale was validated using a different observational protocol than the one used here: children were observed during 15 min of free play indoors and 15 min of free play outdoors [37], whereas in the present study both scales were scored from a single, approximately 40-min, semi-structured observation in a dedicated clinic room using a standard set of materials rather than fully unstructured free play. This procedural difference does not necessarily invalidate the scores obtained, but the administration context used here does not exactly reproduce that of the Turkish adaptation study, and this should be considered when interpreting the results. In addition, the mother’s presence during the observation varied across participants and was not systematically recorded; because a caregiver’s presence can influence a child’s play behavior and engagement, this unrecorded variability should be regarded as a further limitation of the observational procedure. Because a formal log of families approached and declining participation was not maintained during recruitment, the potential for selection bias in this single-center sample cannot be formally evaluated. Similarly, although ASD and comorbid ADHD diagnoses were confirmed from the rehabilitation center’s clinical records rather than relying solely on maternal report, further diagnostic details-including the diagnosing physician’s specialty and the child’s age at diagnosis-were not systematically recorded and could not be reported, and no information is available on whether a differential evaluation was conducted for ADHD in relation to ASD-related manifestations and developmental level. In addition, the specific diagnostic system, criteria, interviews, or instruments originally used to establish these diagnoses are not known to us, and for ADHD specifically it is not known whether the diagnosis incorporated information from multiple informants or evidence of symptoms and functional impairment across different settings; these details were not available in the clinical records we were able to access and are not reconstructed retrospectively here. The “severe neurological, genetic, or psychiatric disorder” exclusion criterion was applied by reviewing each child’s clinical file at the center; children with such a documented diagnosis in addition to ASD were not enrolled. However, a formal operational definition of “severe” beyond the presence of such a diagnosis in the clinical record was not specified in advance, and the number of children excluded on this basis specifically was not logged. Several other potentially relevant confounders were also not measured, including autism symptom severity, cognitive and language level, sleep, broader behavioral problems, maternal psychiatric history, social support, household income, caregiving burden, specific therapy intensity, and current medication use (including for children with comorbid ADHD), any of which could account for some of the associations reported here. In particular, because measures of autism symptom severity, cognitive/language level, and adaptive functioning were not available, associations involving developmental play performance and playfulness in this study cannot be clearly disentangled from these broader, unmeasured developmental characteristics, and should not be interpreted as isolated features of play in the absence of this information. Finally, the multivariable regression model, which included five predictors estimated from 58 participants, was developed and tested within the same sample; it has not been externally or cross-validated, and its predictive stability in other samples remains to be established.

This study found that anxiety symptoms among mothers of children aged 4–6 years with ASD were closely related to children’s play performance. Lower developmental play performance was independently associated with maternal anxiety, whereas maternal employment status, education level, and school type did not show independent associations once clinical variables were considered; an association with comorbid ADHD was also observed but is preliminary, being based on only 10 children, and requires confirmation. Maternal anxiety and play outcomes were each related to sensory-processing patterns in a pattern- and domain-specific rather than a uniform manner. These findings support the importance of considering children’s sensory and play characteristics alongside family-related psychosocial factors in occupational therapy assessment and intervention for ASD. Larger, multicenter, and longitudinal studies are needed to clarify the direction of these relationships and the mechanisms underlying them.

## Figures and Tables

**Table 1:** Inclusion and exclusion criteria.

**Inclusion Criteria**
• Diagnosis of autism spectrum disorder • Age between 4 and 6 years • Receiving services at ATA Special Education and Rehabilitation Center in Istanbul, Türkiye • Mother’s voluntary agreement to participate in the study • Mother’s ability to read and understand Turkish and complete the study questionnaires
**Exclusion criteria**
• Presence of severe neurological, genetic, or psychiatric disorder in addition to autism spectrum disorder • Mother’s cognitive or communication difficulties that could prevent questionnaire completion • Incomplete or incorrectly completed data collection forms • Unwillingness to participate in the study or withdrawal from the study at any stage

**Table 2:** Demographic and clinical characteristics of the participants.

Variable	n (%)/Mean ± SD
Child age, years	5.12 ± 0.77
• 4 years	14 (24.1)
• 5 years	23 (39.7)
• 6 years	21 (36.2)
Sex	
• Female	29 (50.0)
• Male	29 (50.0)
School type	
• Preschool	41 (70.7)
• Primary school	17 (29.3)
Comorbid ADHD	
• Yes	10 (17.2)
• No	48 (82.8)
Previous therapy history	
• Yes	56 (96.6)
• No	2 (3.4)
Maternal age, years	35.95 ± 3.38
Maternal education level	
• Secondary school	1 (1.7)
• High school	16 (27.6)
• University	41 (70.7)
Maternal employment status	
• Employed	32 (55.2)
• Unemployed	26 (44.8)

Values are presented as mean ± standard deviation or n (%). ADHD: attention-deficit/hyperactivity disorder.

**Table 3:** Descriptive statistics of outcome measures and sensory processing scores.

Variable	Mean ± SD	Median (IQR)	Min–Max
Beck Anxiety Inventory	5.16 ± 4.00	3.50 (2.00–8.00)	1–15
Revised Knox Preschool Play Scale	−2.31 ± 4.19	−1.00 (−4.00–1.00)	−16–3
Test of Playfulness	63.91 ± 15.20	68.00 (55.50–75.00)	20–86
Low registration	94.74 ± 15.26	93.00 (85.00–100.75)	70–135
Sensation seeking	129.00 ± 21.53	125.50 (113.00–145.50)	87–175
Sensory sensitivity	80.86 ± 11.22	78.50 (74.00–85.75)	61–110
Sensation avoiding	98.38 ± 16.23	98.00 (84.00–110.00)	73–130
Auditory processing	27.40 ± 5.18	28.00 (24.00–31.75)	18–38
Visual processing	31.09 ± 5.60	30.50 (27.25–35.75)	20–45
Vestibular processing	36.16 ± 7.16	36.00 (30.25–40.75)	24–52
Touch processing	63.36 ± 12.30	61.50 (55.00–75.75)	40–86
Multisensory processing	23.34 ± 4.12	22.00 (21.00–26.00)	15–33
Oral sensory processing	41.64 ± 7.56	40.00 (36.00–48.00)	25–56
Sensory processing related to endurance/tone	27.34 ± 4.51	28.00 (24.00–30.00)	16–36
Modulation related to body position and movement	31.09 ± 6.51	30.00 (26.25–35.00)	19–46
Modulation of movement affecting activity level	21.71 ± 4.30	22.00 (19.00–24.00)	12–32
Modulation of sensory input affecting emotional responses	12.88 ± 2.87	13.00 (11.00–15.00)	6–19
Modulation of visual input affecting emotional responses and activity level	12.33 ± 2.33	12.00 (11.00–13.00)	8–19
Emotional/social responses	38.55 ± 14.37	43.00 (24.50–49.00)	12–61
Behavioral outcomes of sensory processing	30.03 ± 19.99	19.00 (16.00–42.75)	12–78
Items indicating thresholds for response	10.40 ± 4.51	10.00 (8.00–11.00)	5–28

Values are presented as mean ± standard deviation, median (interquartile range), and minimum–maximum. SD: standard deviation; IQR: interquartile range.

## Data Availability

The data presented in this study are available on request from the corresponding author. The data are not publicly available due to privacy and ethical restrictions protecting participant confidentiality.
